# Transepicondylar distance measured on MRI can predict the length of the graft required for different anterior cruciate ligament reconstruction (ACLR) techniques useful for revision surgery

**DOI:** 10.1186/s10195-022-00670-7

**Published:** 2022-10-15

**Authors:** Federica Rosso, Roberto Rossi, Riccardo Faletti, Antonino Cantivalli, Davide Blonna, Davide Edoardo Bonasia

**Affiliations:** 1 Department of Orthopaedics and Traumatology, AO Ordine Mauriziano Hospital, University of Torino, Largo Turati 62, 10128 Turin, Italy; 2 Department of Radiology, AOU “Città Della Salute e Della Scienza” Hospital, University of Torino, Via Genova 3, 10126 Turin, Italy; 3 Università Degli Studi Di Torino, Via Po 8, 10100 Turin, Italy

**Keywords:** Graft length, Transepicondylar distance, ACL revision, Anterolateral ligament (ALL) reconstruction, Lateral extrarticular tenodesis (LET)

## Abstract

**Background:**

The aim of this study is to find a correlation between linear measurements and the graft length required for different anterior cruciate ligament (ACL) revision techniques, to extract formulas to predict required graft length during the preoperative planning.

**Methods:**

At time 0 and 30 days later, two observers measured eight linear distances on standard 2D knee magnetic resonance imaging (MRI), and nine curved distances on 3D MRI sequences, corresponding to different techniques for ACL revision, anatomic anterolateral ligament (ALL) reconstruction, and lateral extrarticular tenodesis (LET). Intra- and interobserver reliability was tested for 2D and 3D measurements. The correlation between 2D and 3D measurements was tested. The 2D measurements with highest repeatability and reproducibility, and with strongest correlation with 3D measurements were used to extract formulas to calculate the graft length from 2D values.

**Results:**

Fifty MRIs acquired with both 2D and 3D sequences were used. The intra- and interobserver reliability of linear 2D measurement was high, with the transepicondylar distance (TD) showing the highest reproducibility and repeatability. The intra- and interobserver reliability of 3D measurements was lower than 2D, but acceptable for all measurements except for ALL reconstruction. The TD showed the strongest correlation with 3D measurements. The formulas extracted to calculate the graft length from the TD proved to be accurate.

**Conclusion:**

Accurate formulas were created to calculate the graft length needed for different ACL revision techniques and ALL reconstruction/LET techniques from TD. These formulas can be used during preoperative planning of ACL revision cases.

## Introduction

Anterior cruciate ligament reconstruction (ACLR) is one of the most common orthopedic procedures [1, 2]. A better understanding of the anatomy of the anterior cruciate ligament (ACL= [3] together with the anatomy and biomechanics of the anterolateral structures of the knee [4] has recently led to changes and improvements in the surgical techniques for anatomic ACLR. However, suboptimal results with persistent residual instability and ACLR failure have been described in up to 10% of cases [5–7], with a risk of ACL re-rupture ranging between 6% and 32% [8, 9]. During ACLR revision, the surgeon has to deal with different issues compared with primary reconstruction, including: (1) the management of previous malpositioned or widened tunnels (technical errors are still the most common cause for ACLR re-rupture [10]); (2) the limited availability of autologous ACL grafts and possible need for allografts; and (3) the treatment of concomitant conditions such as malalignment (in the coronal and sagittal planes), meniscal or chondral injuries, or previously overlooked associated ligamentous deficiencies [11]. In some ACLR revision cases, a two-stage revision (with tunnel bone grafting as a first stage) is the only option available. In other cases, a single stage ACLR revision can be performed with standard anatomical ACLR techniques or “unconventional” anatomical or non-anatomical techniques (i.e., Marcacci, Saragaglia, Yamaguchi techniques) [12–14]. These techniques were initially described for primary ACLR, with good reported outcomes [12–14], but they can also be the single-stage solution for specific ACLR revision cases because they allow for an easy management of malpositioned or widened femoral tunnels (the graft is placed over the top on the femur or in a femoral tunnel drilled outside-in, depending on the technique); femoral fixation is not an issue for the surgeon is some of these techniques; most of these techniques involve a lateral plasty (according to some data, ACLR revision is one of the indications for anterolateral ligament reconstruction or anterolateral tenodesis/plasty) [4]; and they can be performed with autograft or allografts.

The length of the graft needed to perform these procedures is significantly longer than in standard ACLR, and knowing it preoperatively can be useful to switch to another technique when the harvested hamstrings (HS) are too short, to harvest the correct length of fascia lata autograft (i.e., in Yamaguchi technique [13]), and to request an adequately long allograft. Furthermore, knowing preoperatively the length of the intrarticular portion of the graft and the length of the tunnels can prevent a graft-tunnel mismatch in case the ACLR revision is performed with a standard bone patella tendon bone (BPTB) technique.

Measuring the length of the graft needed on standard 2D magnetic resonance imaging (MRI) is not possible, but high-quality isotropic 3D MRI sequences should be obtained for a 3D reconstruction and multiplanar reformatting [15]. However, 3D isotropic sequences are not commonly performed due to the following reasons: (1) 3D MRI requires a longer acquisition time compared with 2D sequences; (2) owing to the longer acquisition time, 3D isotropic sequences are more susceptible to motion artifacts; and (3) the sensitivity of 3D MRI for meniscal and ACL injuries seems to be lower than 2D MRI because of image blurring, decreased in-plane resolution, and suboptimal soft-tissue contrast [16, 17]. For these reasons, a direct measurement of the graft length required for ACLR revision techniques on 3D MRI is not possible.

The aim of this study was to obtain formulas to calculate the length of the graft required for different ACLR techniques (useful in the revision setting), from simple and linear 2D measurements on 2D MRI. However, the following steps have to be performed before obtaining such formulas: (1) test the intra and interobserver reliability of linear 2D measurements (i.e., transepicondylar distance, proximal tibial width, etc.) on standard 2D MRI sequences; (2) test the intra and interobserver reliability of complex/curved 3D measurements (i.e., length of the graft required to perform a Marcacci or Yamaguchi technique, etc.) on 3D MRI; and (3) investigate the correlation between linear 2D MRI measurements with 3D measurements (that are the length of the graft required for different ACL revision/reconstruction techniques) to find the 2D measurement with the higher correlation, which will be used to extract the formulas. The authors’ hypotheses are (1) there is a correlation between some 2D measurements and 3D measurements, and (2) creating formulas to calculate the length of the required graft based on 2D measurement is possible, and would be useful for ACLR revision preoperative planning.

## Methods

This study was approved by the local Ethical Committee (University of BLIND FOR REVIEW, Protocol number BLIND FOR REVIEW).

Fifty healthy volunteers younger than 55 years of age were enrolled (from January 2018 to Mar 2019), after signing an informed consent for participation in this study. Exclusion criteria included: (1) presence of arthritis; (2) congenital/hereditary diseases causing articular deformities (i.e., spondyloepiphyseal dysplasia, congenital patellar dislocation, etc.); (3) previous knee surgeries; (4) chronic inflammatory diseases; (5) presence of hardware in the knee; and (6) previous tibial, femoral, or patellar fractures. Patient demographics were recorded, including age, sex, height, weight, and body mass index (BMI).

All images were acquired with a 1.5 T MRI scanner (Philips Achieva 1.5 T MRI System; Philips Medical Systems, Best, the Netherlands) and an eight-channel SENSE knee coil. Routine 2D (sagittal T1w-TSE and T2w-TSE Fat Suppression images, axial T2w-TSE images and coronal Gradient Echo T2w images) and 3D Turbo Spin Echo (TSE) T2 weighted images with isotropic voxel (Volume Isotropic Turbo Spin echo Acquisition, VISTA Philips, 0.6 × 0.6 × 0.6 mm) were acquired in each patient. The acquisition time was 5–10 min for the entire 2D MRI protocol and 5–10 min for the T2 VISTA 3D sequence. The sagittal source images from the 3D TSE technique were used to create sagittal, coronal, and axial reformatted images of the knee joint. The reformatted images were used for the 3D TSE assessment of the knee. The post-processing of the 3D TSE sequence was performed by a radiologist on a Philips Achieva MRI workstation (Extended MR Workspace; Philips Medical Systems) after acquisition of the images.

Two sports medicine fellowship trained orthopedic surgeons (initial blind for review with DEB and FR) performed the 2D and 3D measurements at time 0 (T0) and after 30 days (T1) on the Philips Achieva MRI workstation (Extended MR Workspace; Philips Medical Systems).

### 2D- Measurements

To decide the 2D measurements to be included in the study, two sports medicine fellowship trained orthopedic surgeons (initial blind for review with DEB and FR) were asked the following question:

Based on the recent literature and your experience, which 2D measurements having the following characteristics would you include in this study?

- Morphological measurements of the knee joint (bone or soft tissue) that can potentially affect the length of the graft required for different ACLR revision techniques
- Measurements obtainable on standard 2D MRI protocols
- Measurements easy to perform in the clinical practice (with simple or no instructions to be provided to clinicians)
- Include one or more measurements potentially affected by both tibial and femoral dimensions

**Fig. 1:**
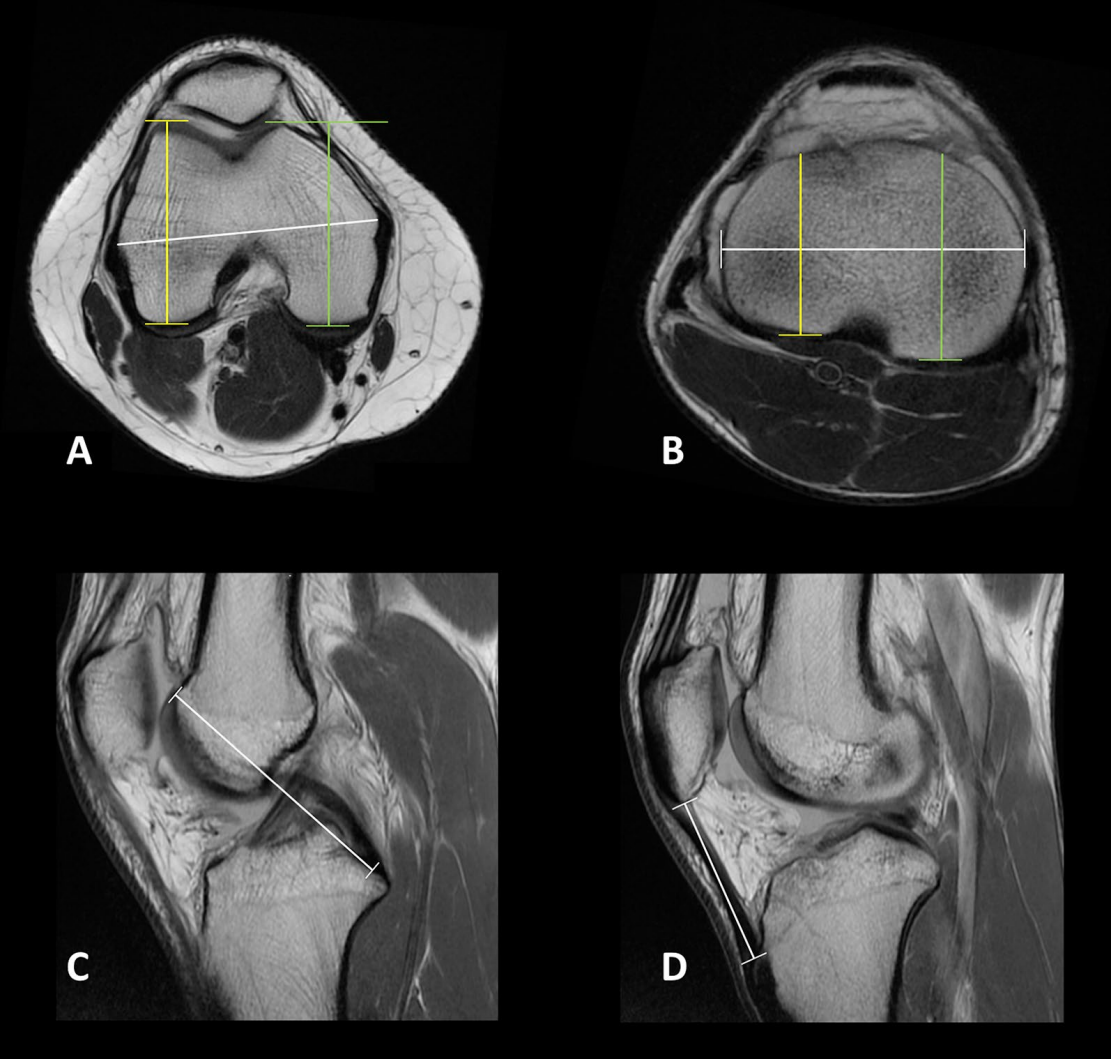
2D measurements on T1-weighted MRI sequences: **A** Axial view of the femur: transepicondylar Distance (TD, white line), medial femoral condyle anteroposterior (AP) dimension (MFAP, green line), and lateral femoral condyle AP dimension (LFAP, yellow line), measured on axial cut. **B** Axial view of the tibia: proximal tibia medio-lateral dimension (PTML, white line), medial tibial plateau AP dimension (MTAP, green line), lateral tibial plateau AP dimension (LTAP, yellow line). **C** Sagittal view: posterior cruciate ligament and trochlear cartilage distance (PCLTC, white line). **D** Sagittal view: patellar tendon length (PTL, white line)

After discussion, the two surgeons agreed on the following measurements to be included [18] (Fig. 1):

1. Transepicondylar distance (TD): defined as the longest distance between the apices of the medial and lateral femoral epicondyles in the mediolateral (ML) axis, measured on axial cuts.
2. Medial femoral condyle anteroposterior (AP) dimension (MFAP): defined as the longest dimension of the medial femoral condyle in the AP axis, measured on axial cuts.
3. Lateral femoral condyle AP dimension (LFAP): defined as the longest dimension of the lateral femoral condyle in the AP axis, measured on axial cuts.
4. Proximal tibia medio-lateral dimension (PTML): defined as the longest mediolateral dimension of the proximal tibial in the ML axis, measured on proximal axial cuts just distal to the menisci.
5. Medial tibial plateau AP dimension (MTAP): defined as a segment drawn perpendicular to PTML and passing through the most posterior point of the medial tibial condyle in the AP axis, measured on proximal axial cuts just distal to the menisci.
6. Lateral tibial plateau AP dimension (LTAP): defined as a segment drawn perpendicular to PTML and passing through the most posterior point of the lateral tibial condyle in the AP axis, measured on proximal axial cuts just distal to the menisci
7. Posterior cruciate ligament (PCL) and trochlear cartilage distance (PCLTC): defined as an oblique distance between the center of the PCL tibial insertion and the most anterior and proximal point of the trochlear cartilage, measured on sagittal cuts.
8. Patellar tendon length (PTL): defined as an oblique distance between the inferior pole of the patella and the most proximal part of the patellar tendon insertion on the tibial tuberosity, measured on sagittal cuts.

The observers were intentionally not instructed regarding the weighting of the sequence for 2D measurements, for possible subsequent application in the clinical setting.

### 3D Measurements

The 3D measurements were performed on the 3D MRI sequences (Turbo Spin Echo T2 weighted 3D MRI with isotropic voxel, VISTA Philips) by the same observers at time 0 (T0) and after 30 days (T1). The 3D measurements corresponded to different ACLR revision techniques and anatomic anterolateral ligament (ALL) reconstruction or lateral extrarticular tenodesis (LET).

**Fig. 2:**
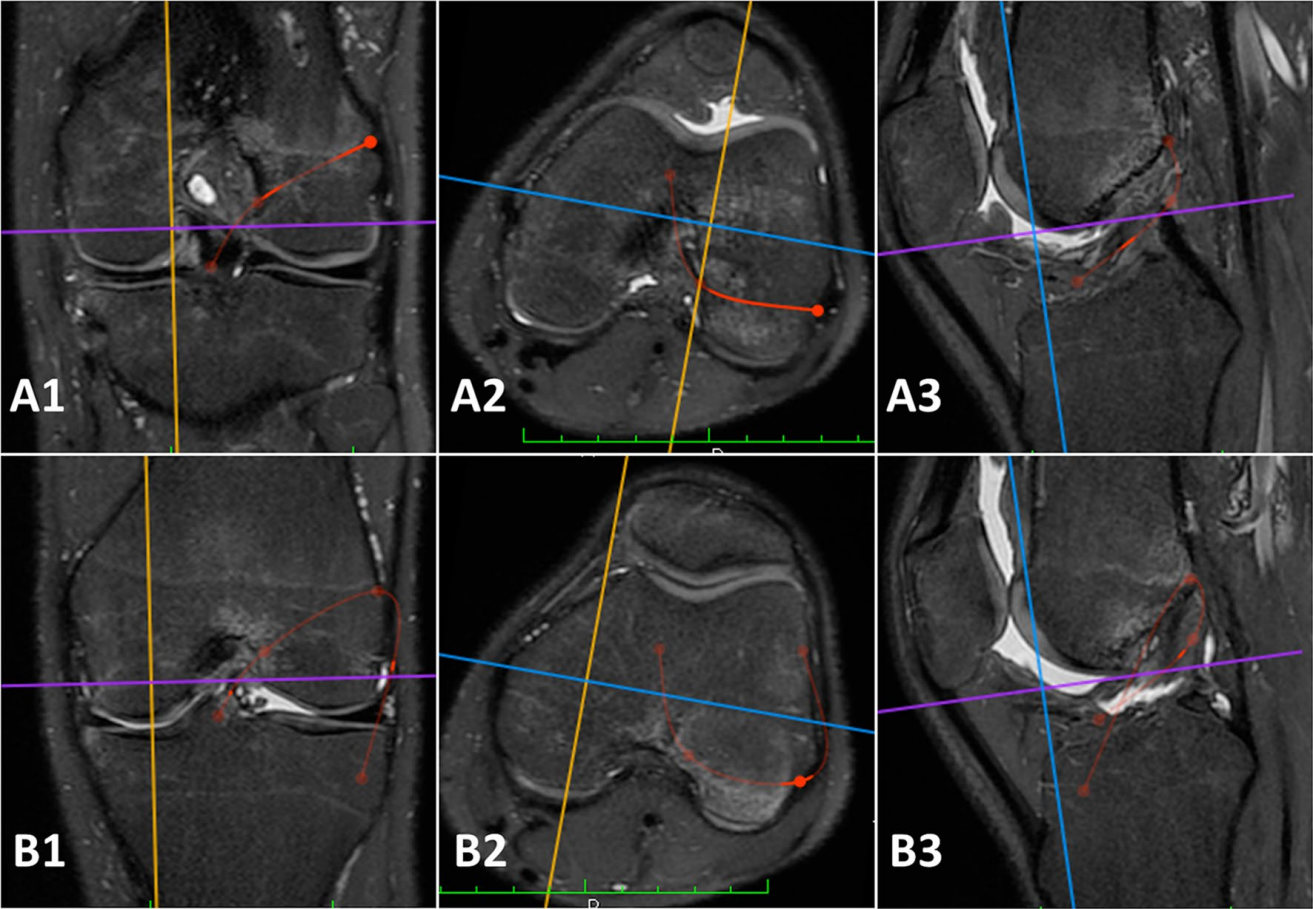
3D measurements performed on the 3D MRI sequences (Turbo Spin Echo T2 weighted 3D MRI with isotropic voxel). **A** Red lines: single bundle outside-in anatomic ACL reconstruction (SBOI) defined as the sum of intrarticular length of the graft (IAGL) and Outside-in femoral tunnel (OIFT). Particularly, A1 is the coronal view, A2 axial view, and A3 sagittal view of a left knee with simulation of the same reconstruction. **B** Red lines: Saragaglia and Yamaguchi technique (SYT) defined as the sum intrarticular length of the graft (IAGL), outside-in femoral tunnel (OIFT) and modified Lemaire lateral extrarticular tenodesis, anatomical on the femur (ALET). Particularly, B1 is the coronal view, B2 is the axial view, and B3 is the sagittal view of a left knee with simulation of the same reconstruction

**Fig. 3:**
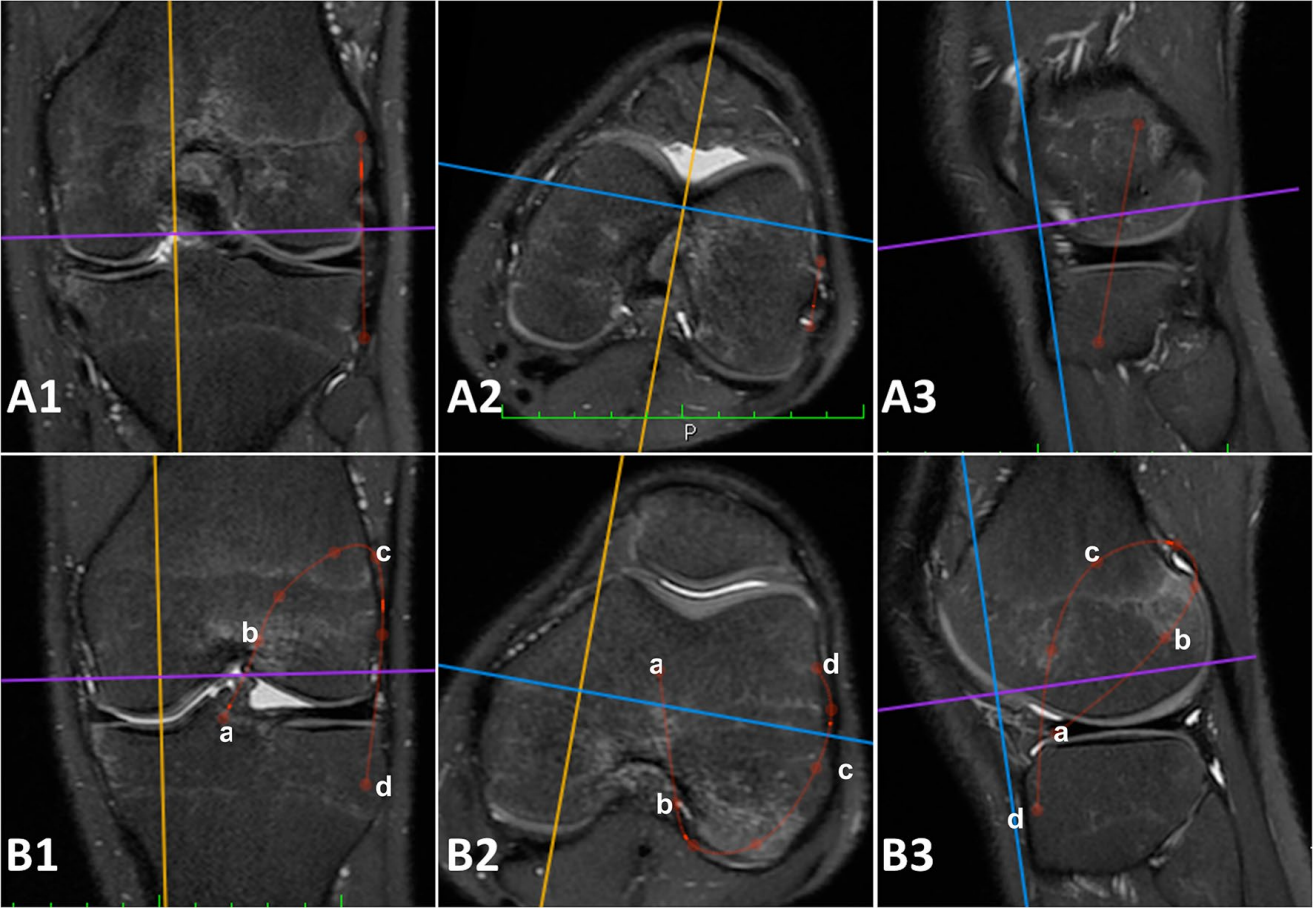
3D measurements performed on the 3D MRI sequences (Turbo Spin Echo T2 weighted 3D MRI with isotropic voxel). **A** Red lines: anatomic anterolateral ligament (ALL) reconstruction defined as the distance from a point located 5 mm proximal and 5 mm posterior to the apex of the lateral femoral epicondyle and a tibial point equidistant between the center of the Gerdy’s tubercle and the anterior margin of the fibular head, 9.5 mm distal to the joint line. Particularly, A1 is the coronal view, A2 is the axial view, and A3 is the sagittal view of a left knee with simulation of the same reconstruction. **B** Red lines: Marcacci technique (MT) defined as defined as the ACL graft path of the Marcacci technique, passing through **a** the center of the tibial ACL footprint; **b** the over the top femoral position; **c** the distal aspect of the proximal ridge of the femur at the diaphyseal-metaphyseal junction; **d** the Gerdy’s tubercle. Particularly, B1 is the coronal view, B2 is the axial view, and B3 is the sagittal view of a left knee with simulation of the same reconstruction

The measurements included (Figs. 2 and 3) were:

1. Intrarticular length of the graft (IAGL): defined as the distance from the center of the tibial ACL footprint to the center of the femoral footprint
2. Outside-in femoral tunnel (OIFT) length: defined as the distance from the center of the femoral ACL footprint to a point located 5 mm proximal and 5 mm posterior to the apex of the lateral femoral epicondyle.
3. Single bundle outside-in anatomic ACL reconstruction, without tibial tunnel (SBOI) length: defined as the sum of measurements one and two. It should represent the total length of the reconstruction.
4. Anatomic anterolateral ligament (ALL) reconstruction length according to Laprade et al. (without tibial and femoral half sockets) [19]: defined as the distance from a point located 5 mm proximal and 5 mm posterior to the apex of the lateral femoral epicondyle and a tibial point equidistant between the center of the Gerdy’s tubercle and the anterior margin of the fibular head, 9.5 mm distal to the joint line. It is representative also of the length of the iliotibial band (ITB) graft required to perform this technique, without considering the amount of graft in the socket.
5. Modified Lemaire lateral extrarticular tenodesis length, anatomical on the femur (ALET): defined as the distance from the center of the Gerdy’s tubercle to a point located 5 mm proximal and 5 mm posterior to the apex of the lateral femoral epicondyle, passing under the lateral collateral ligament, and representing the length of the required graft [20].
6. Length of Saragaglia and Yamaguchi technique, without tibial tunnel (SYT): defined as the sum of measurements one, two, and five. In the original Saragaglia technique the hamstrings were harvested (tripled semitendinosus, single gracilis) and left attached, then passed through a standard tibial tunnel. The femoral tunnel was performed in an outside-in manner inferior to the proximal insertion of the lateral collateral ligament (LCL). The graft is fixed with absorbable screws in the femoral and tibial tunnel. A lateral skin incision is performed at the Gerdy’s tubercle and the remaining portion of the graft is passed under the fascia and fixed to the tubercle with anchor or nonabsorbable stitches with the knee at 30° of flexion and the foot in neutral position. The Yamaguchi technique is similar, but it is performed with a strip of ITB left attached to the Gerdy’s tubercle, passed under the fascia and then into a femoral and tibial tunnel performed as in the Saragaglia technique [13, 14]. In both the cases, 3D measurements represents the length of the required graft without the tibial tunnel.
7. Over the top femoral position ACL reconstruction length, without tibial tunnel (OTT): defined as the ACL graft path from a point located just distal to the proximal ridge of the distal femur at the diaphyseal-metaphyseal junction to a point located in the center of the tibial ACL footprint [21]. It would be representative of the length of the required graft.
8. Marcacci technique length, without tibial tunnel (MT): defined as the ACL graft path of the Marcacci technique, passing through (a) the center of the tibial ACL footprint; (b) the over the top femoral position; (c) the distal aspect of the proximal ridge of the femur at the diaphyseal-metaphyseal junction; (d) the Gerdy’s tubercle [12]. Particularly, the Marcacci technique is a combined reconstruction originally performed using hamstrings left attached to the tibia. The graft is then passed into a standard tibial tunnel, then in the over-the-top position on the femur, behind the lateral femoral condyle and fixed on the lateral cortex of the lateral femoral condyle with two staples. The remaining portion of the graft is passed under the fascia and fixed at the Gerdy’s tubercle with one staple.
9. Modified Lemaire LET procedure length, non-anatomical on the femur (NALET) according to Spencer et al. (more proximal and posterior fixation on the femur) [22]: defined as measurement 8 minus measurement 7, and representative of the required graft length. The tibial tunnel measurement was intentionally excluded from all 3D measurements because its length is not a fixed value and can be adjusted intraoperatively, by varying the location of the distal aperture of the tunnel itself.

### Statistical analysis

Data were reported with mean, standard deviation, and/or ranges. The normality of the distribution of the measurements was tested with the D’Agostino-Pearson test. The intraobserver reliability was tested with the Pearson correlation index (*r*) in case of normal distribution or the Spearman’s rank correlation coefficient (*rho*) in case of not normal distribution, together with the Cronbach’s *alpha* coefficient. The interobserver reliability was tested with the Cronbach’s *alpha* coefficient, the Kappa index, and the intraclass correlation coefficient (ICC). In addition, the correlation between the 2D measurements and the 3D measurements was tested with the Pearson’s correlation index and Spearman’s rank correlation coefficient (according to the normality of the distribution). The 2D measurements with highest intra and interobserver reliability and with the strongest correlation with 3D measurements (length of the different reconstruction techniques) were used to extract formulas to calculate the 3D measurements from 2D values. The formulas were obtained with linear regression. In addition, the accuracy of the curved measurements obtained with the formulas was compared with the same measurements performed on the 3D MRI and the difference between the groups was tested with a paired *t*-test and Bland Altman plots. Statistical analysis was performed with MedCalc Statistical Software version 16.4.3 (MedCalc Software, Ostend, Belgium).

Patients or the public were not involved in the design, conduct, reporting, or dissemination plans of our research.

## Results

### Patients’ demographics

Fifty patients (50 knees) were included in the study, 18 (36%) were women, 32 (64%) were men, and the mean age was 28.1 years (SD 6.14, range 20–50 years). The mean age was 27.89 years (SD 5.95, range 22–43 years) for women, and 28.22 years (SD 6.34, range 20–50 years) for men. The average height was 179 cm (SD 6.67, range 166–194 cm) for men, 169 cm (SD 5.95 range 158–176 cm) for women, and 175 cm (SD 8.1, range 158–194 cm) for the total population. The right knee was studied in 50% of the study group.

Table 1 summarizes the means and standard deviations (SD) of the 2D and 3D measurements for the two observers at time 0 and time 1 (30 days after T0). In the population studied, data showed that the difference in the graft length between small and large knees could be significant; this difference was about 2 cm for shorter revision techniques (i.e., over the top femoral placement and single bundle out-in femoral tunnel ACLR revision) and about 4 cm for longer revision techniques (i.e., Saragaglia, Yamaguchi, Marcacci).

**Table 1:** Summary of the 2D and 3D measurements in mm for the two observers at Time 0 and Time 1 (30 days after Time 0)

Parameter measured (mm)	Observer 1				Observer 2				Observer 1 (T0 + T1) + Observer 2 (T0 + T1)		
	Time 0		Time 1		Time 0		Time 1				
	Mean	SD	Mean	SD	Mean	SD	Mean	SD	Mean	SD	Range
2D measurements											
Transepicondylar distance (TD)	82.6	6.5	82.5	6.3	82.9	6.1	83.2	6.4	82.8	6.3	71.9–94-9
Medial Femoral condyle AP dimension	60.8	3.7	60.1	3.7	61.0	4.0	61.1	6.0	60.8	3.7	54.4–71.1
Lateral Femoral condyle AP dimension	61.7	4.2	61.6	4.2	62.0	4.2	62.1	4.6	61.9	4.2	54.5–70.4
Proximal tibia ML dimension	74.4	5.8	75.2	5.7	74.9	5.9	75.6	5.6	75.0	5.6	65.3–85.4
Medial tibial plateau AP dimension	47.0	3.7	46.4	3.8	52.5	4.2	48.5	4.6	48.6	3.8	42.3–58.7
Lateral tibial plateau AP dimension	45.1	3.8	45.3	3.9	45.9	4.0	44.8	3.8	45.3	3.7	39.6–52.2
Tibial PCL insertion-trochlearcartilage distance	81.5	5.8	81.4	6.0	80.3	5.4	82.5	6.0	81.4	5.7	71–97.7
Patellar tendon length	47.8	6.3	46.5	5.8	45.2	3.9	46.1	4.5	46.4	4.6	37.7–58.4
3D measurements											
Intrarticular length of ACL graft	37.5	4.2	37.5	3.8	39.9	3.5	38.6	4.2	38.3	3.5	31.6–45.4
Outside-in femoral tunnel length	34.1	3.5	33.2	3.0	34.8	3.0	35.1	3.0	34.3	2.9	29.2–40.2
Outside-in anatomic ACL reconstruction	69.4	5.9	70.9	5.7	74.1	5.9	73.7	6.6	72.0	5.7	61–81.6
Saragaglia/Yamaguchi techniques	125.6	11.2	123.5	9.4	120.6	8.1	121.9	9.6	122.9	9.0	107.1–142.2
Anatomic Anterolateral ligament reconstruction	52.6	5.3	51.3	4.3	45.6	3.3	48.2	4.6	49.4	3.5	42.9–58.4
Lateral extrarticular tenodesis (anatomical femur)	54	5.3	52.6	4.4	46.5	3.5	48.2	4.6	50.3	3.6	43.6–59.1
Lateral extrarticular tenodesis (non-anatomical femur)	69.9	6.9	73.4	5.8	65.5	5.3	70.4	4	69.8	4.6	61.7–78
Over the top femoral position ACL reconstruction	74.0	6.6	76.9	6.0	85.1	6.7	76.8	6.5	78.2	5.9	67.3–89
Marcacci technique	139.5	28.6	150.3	10.7	150.5	10.8	147.2	9.8	146.9	13.2	129.7–166.4

The last column includes the mean and SD obtained from the sum of Observer 1 (Time0) + Observer 1 (Time 1) + Observer 2 (Time0) + Observer 2 (Time 1)

*SD* Standard deviation, *AP* anteroposterior, *ML* mediolateral, *PCL* posterior cruciate ligament, *ACL* anterior cruciate ligament

### Intraobserver reliability

The intr-observer reliability (Pearson’s or Spearman’s correlation according to the normality of the data distribution and Cronbach’s alpha) for the 2D and 3D measurements is summarized in Table 2. Among the 2D measurements, the highest intraobserver reliability was found for the transepicondylar distance (TD) for both observers. As presented in Table 2, 2D measurements were more repeatable than 3D. However, the intraobserver reliability was acceptable (greater than 0.6 in all the cases, with most of the measurement showing an intraobserver reliability greater than 0.8) in all measurements.

**Table 2:** Intraobserver reliability (Pearson's or Spearman's correlation according to the normality of the data distribution and Cronbach's alpha) for the 2D and 3D measurements

Intraobserver reliability	Pearson’s correlation index				Spearman’s rank correlation coefficient				Cronbach's alpha			
	Observer 1		Observer 2		Observer 1		Observer 2		Observer 1		Observer 2	
	*r*	*p*-Value	*r*	*p*-Value	*rho*	*p*-Value	*rho*	*p*-Value	*Alpha*	IC95%	*Alpha*	IC95%
2D measurements												
Transepicondylar distance (TD)	–	–	–	–	0.989	< 0.0001	0.992	< 0.0001	0.9942	0.9906	0.9946	0.9913
Medial femoral condyle AP dimension	0.8989	< 0.0001	0.9321	< 0.0001	–	–	–	–	0.9468	0.9144	0.9649	0.9436
Lateral femoral condyle AP dimension	0.9715	< 0.0001	0.8697	< 0.0001	–	–	–	–	0.9856	0.9768	0.9303	0.8880
Proximal tibia ML dimension	–	–	–	–	0.967	< 0.0001	0.925	< 0.0001	0.9835	0.9735	0.9369	0.8986
Medial tibial plateau AP dimension	0.8525	< 0.0001	0.8250	< 0.0001	–	–	–	–	0.9204	0.8721	0.9041	0.8458
Lateral tibial plateau AP dimension	–	–	–	–	0.913	< 0.0001	0.904	< 0.0001	0.9087	0.8533	0.9514	0.9219
Tibial PCL insertion-trochlearcartilage distance	0.9549	< 0.0001	0.9515	< 0.0001	–	–	–	–	0.9769	0.9629	0.9752	0.9601
Patellar tendon length	–	–	0.8236	< 0.0001	0.735	< 0.0001	–	–	0.8925	0.8273	0.9033	0.8445
3D measurements												
Intrarticular length of ACL graft	0.6103	< 0.0001	0.7075	< 0.0001	–	–	–	–	0.758	0.6111	0.8287	0.7246
Outside-in femoral tunnel length	0.8221	< 0.0001	–	–	–	–	0.915	< 0.0001	0.9023	0.843	0.9433	0.9089
Outside-in anatomic ACL reconstruction	0.8918	< 0.0001	–	–	–	–	0.869	< 0.0001	0.9428	0.9081	0.9206	0.8723
Saragaglia/Yamaguchi techniques	0.8654	< 0.0001	0.7654	< 0.0001	–	–	–	–	0.9278	0.884	0.7699	0.6302
Anatomic anterolateral ligament reconstruction	0.6953	< 0.0001	0.2675	0.0603	–	–	–	–	0.8202	0.7111	0.4221	0.0712
Lateral extrarticular tenodesis (anatomical femur)	0.6911	< 0.0001	0.2834	0.0461	–	–	–	–	0.8173	0.7064	0.4282	0.1025
Lateral extrarticular tenodesis (non-anatomical femur)	0.5914	< 0.0001	0.6043	< 0.0001	–	–	–	–	0.7432	0.5973	0.7533	0.6035
Over the top femoral position ACL reconstruction	0.7718	< 0.0001	0.7601	< 0.0001	–	–	–	–	0.8712	0.793	0.8637	0.7809
Marcacci technique	–	–	–	–	0.859	< 0.0001	0.800	< 0.0001	0.8180	0.7075	0.8823	0.8108

*AP* anteroposterior, *ML* mediolateral, *PCL* posterior cruciate ligament, *ACL* anterior cruciate ligament

### Interobserver reliability

The interobserver reliability tested with intraclass correlation coefficient (ICC), Kappa Index, and Cronbach's alpha for the 2D and 3D measurements is presented in Table 3.

**Table 3:** Interobserver reliability tested with intraclass correlation coefficient (ICC), Kappa Index, and Cronbach's alpha for the 2D and 3D measurements

Interobserver reliability	Intraclass correlation coefficient (ICC)				K index			Cronbach’s alpha	
	*Single measures*	*CI 95%*	*Average measures*	*CI95%*	*Obs 1 (T1) and Obs 2 (T1)Kappa*	*SD*	*CI 95%*	*Obs 1 (T1) and Obs 2 (T1) Alpha*	*CI 95%*
2D measurements									
Transepicondylar distance (TD)	0.9878	0.9786–0.9931	0.9939	0.9892–0.9965	0.864	0.019	0.827–0.902	0.9939	0.9902
Medial femoral condyle AP dimension	0.877	0.7929–0.9283	0.9344	0.8845–0.9628	0.677	0.049	0.580–0.774	0.9369	0.8986
Lateral femoral condyle AP dimension	0.8882	0.8110–0.9300	0.9408	0.8956–0.9664	0.772	0.042	0.490–0.853	0.9427	0.9079
Proximal tibia ML dimension	0.9788	0.9630–0.9879	0.9893	0.9811–0.9939	0.858	0.02	0.820–0.897	0.9894	0.9829
Medial tibial plateau AP dimension	0.8533	0.7552–0.9140	0.9208	0.8605–0.9551	0.535	0.062	0.414–0.657	0.9291	0.8861
Lateral tibial plateau AP dimension	0.8955	0.8228–0.9393	0.9448	0.9028–0.8687	0.647	0.038	0.572–0.723	0.9449	0.9114
Tibial PCL insertion-trochlearcartilage distance	0.9406	0.8977–0.9659	0.9694	0.9461–0.9826	0.724	0.041	0.0645–0.804	0.9694	0.9509
Patellar tendon length	0.7314	0.5706–0.8381	0.8448	0.7266–0.9119	0.515	0.067	0.383–0.647	0.8603	0.7754
3D measurements									
Intrarticular length of ACL graft	0.771	0.6379–0.8671	0.8745	0.7789–0.928	0.535	0.066	0.405–0.664	0.8770	0.8022
Outside-in femoral tunnel length	0.8475	0.7461–0.9105	0.9175	0.8546–0.9532	0.457	0.053	0.354–0.560	0.9175	0.8674
Outside-in anatomic ACL reconstruction	0.9121	0.8501–0.9491	0.954	0.9190–0.9739	0.585	0.048	0.492–0.678	0.9590	0.9340
Saragaglia/Yamaguchi techniques	0.8267	0.7136–0.8978	0.9051	0.8329–0.9462	0.619	0.059	0.503–0.735	0.9053	0.8477
Anatomic anterolateral ligament reconstruction	0.4244	0.1682–0.6270	0.5959	0.2880–0.7707	0.271	0.071	0.131–0.410	0.5967	0.3517
Lateral extrarticular tenodesis (anatomical femur)	0.6268	0.4242–0.7696	0.7706	0.5957–0.8698	0.342	0.066	0.213–0.470	0.798	0.6753
Lateral extrarticular tenodesis (non-anatomical femur)	0.8831	0.8028–0.9319	0.9379	0.8906–0.9648	0.638	0.047	0.546–0.730	0.9396	0.9030
Over the top femoral position ACL reconstruction	0.8517	0.7526–0.9131	0.9199	0.8589–0.9545	0.641	0.047	0.546–0.730	0.9218	0.8743
Marcacci technique	0.8831	0.8028–0.9319	0.9379	0.8906–0.9648	0.638	0.047	0.546–0.730	0.9396	0.9030

The second measurement (T1) of each observer was used for every test

*CI* Confidence interval, *AP* anteroposterior, *ML* mediolateral, *PCL* posterior cruciate ligament, *ACL* anterior cruciate ligament

The second measurement (T1) of each observer was used for every test. Among the 2D measurements, the highest interobserver reliability was found for the transepicondylar distance.

As expected, 2D measurements showed higher interobserver reliability than 3D measurements. However, the reproducibility was acceptable for all 2D and 3D measurements, except for anatomic anterolateral ligament (ALL) reconstruction.

### Correlation between 2D and 3D measurements

The correlation between 2D measurements (independent variables) and 3D measurements (dependent variables) was analyzed with Pearson's or Spearman's correlation tests (based on the normality of the data distribution) and is presented in Table 4.

**Table 4:** Correlation between 2D measurements (independent variables) and 3D measurements (dependent variables)

Correlation between 2 and 3D variables	Dependent variables (3D measurements)																	
	Intrarticular length of ACL graft		Outside-in femoral tunnel length		Outside-in anatomic ACL reconstruction		Saragaglia/Yamaguchi techniques		Anatomic anterolateral ligament reconstruction		lateral extrarticular tenodesis (anatomical femur)		lateral extrarticular tenodesis (non-anatomical femur)		Over the top femoral position ACL reconstruction		Marcacci technique	
Independent variables (2D measurements)	Coeff	*p*-value	Coeff	*p*-value	Coeff	*p*-value	Coeff	*p*-value	Coeff	*p*-value	Coeff	*p*-value	Coeff	*p*-value	Coeff	*p*-value	Coeff	*p*-value
Transepicondylar distance (TD)	**0.85**	** < 0.0001**	**0.91**	** < 0.0001**	**0.94**	** < 0.0001**	**0.92**	** < 0.0001**	**0.73**	** < 0.0001**	0.74	< 0.0001	**0.81**	** < 0.0001**	**0.91**	** < 0.0001**	**0.9**	** < 0.0001**
Medial Femoral condyle AP dimension	0.75	< 0.0001	**0.8**	** < 0.0001**	**0.85**	** < 0.0001**	0.85	< 0.0001	0.75	< 0.0001	0.75	< 0.0001	0.8	< 0.0001	0.75	< 0.0001	0.86	< 0.0001
Lateral Femoral condyle AP dimension	0.8	< 0.0001	**0.76**	** < 0.0001**	**0.84**	** < 0.0001**	0.85	< 0.0001	0.74	< 0.0001	0.73	< 0.0001	0.86	< 0.0001	0.82	< 0.0001	0.87	< 0.0001
Proximal tibia ML dimension	**0.82**	** < 0.0001**	**0.85**	** < 0.0001**	**0.89**	** < 0.0001**	**0.9**	** < 0.0001**	**0.76**	** < 0.0001**	0.76	< 0.0001	**0.83**	** < 0.0001**	**0.88**	** < 0.0001**	**0.89**	** < 0.0001**
Medial tibial plateau AP dimension	0.8	< 0.0001	**0.81**	** < 0.0001**	0.88	< 0.0001	0.85	< 0.0001	0.7	< 0.0001	0.71	< 0.0001	**0.8**	** < 0.0001**	0.88	< 0.0001	**0.9**	** < 0.0001**
Lateral tibial plateau AP dimension	**0.8**	** < 0.0001**	**0.85**	** < 0.0001**	**0.89**	** < 0.0001**	**0.89**	** < 0.0001**	0.75	< 0.0001	0.75	< 0.0001	**0.83**	** < 0.0001**	0.9	< 0.0001	**0.89**	** < 0.0001**
Tibial PCL insertion-trochlearcartilage distance	**0.8**	** < 0.0001**	**0.8**	** < 0.0001**	**0.84**	** < 0.0001**	**0.85**	** < 0.0001**	0.76	< 0.0001	0.73	< 0.0001	**0.8**	** < 0.0001**	**0.86**	** < 0.0001**	**0.87**	** < 0.0001**
Patellar tendon length	0.39	0.0052	**0.52**	**0.0001**	**0.52**	**0.0001**	0.5	0.0002	0.47	0.0006	0.46	< 0.0001	0.55	0.0001	0.51	0.0001	0.51	0.0001
Independent variables (Morphometric data)																		
Patient's height	0.68	< 0.0001	**0.73**	** < 0.0001**	**0.78**	** < 0.0001**	0.76	< 0.0001	0.68	< 0.0001	0.66	< 0.0001	0.78	< 0.0001	0.76	< 0.0001	0.77	< 0.0001

Pearson’s or Spearman’s correlation test were used according to the normality of the data distribution. In addition, the patients’ height was tested as well as an independent variable for possible correlation with 3D measurements

The coefficients and *p*-values are bold when the Spearman’s test was used

*Coeff* coefficients, *AP* anteroposterior, *ML* medi iolateral, *PCL* posterior cruciate ligament, *ACL* anterior cruciate ligament

In addition, the patients’ height was tested as well as an independent variable for possible correlation with 3D measurements.

All independent variables, except for patellar tendon length and patient’s height, were strongly correlated with the dependent variables (3D measurements). The transepicondylar distance (TD) showed the strongest correlation with most of the dependent variables.

### Linear regression and mathematical formulas

Simple linear regression was used to develop mathematical formulas able to calculate the 3D measurements (Y variable) from the transepicondylar distance (X variable). The transepicondylar distance (TD) was chosen among all the other 2D measurements because it had the highest repeatability, reproducibility, and strongest correlation with 3D measurements. A linear correlation was found between the transepicondylar distance and all 3D measurements.

The formulas obtained with the linear regression were:

- Intrarticular length of ACL graft (IAGL) = 0.13 + (0.46 × TD)
- Outside-in femoral tunnel length (OIFT) = 0.62 + (0.42 × TD)
- Outside-in anatomic ACL reconstruction (SBOI) length = 2.26 + (0.84 × TD) + tibial tunnel
- Length of Saragaglia/Yamaguchi techniques (SYT) = 15.09 + (1.3 × TD) + tibial tunnel
- Lateral extrarticular tenodesis anatomical femur (ALET) length = 15.49 + (0.42 × TD)
- Lateral extrarticular tenodesis non-anatomical femur (NALET) length = 21.23 + (0.59 × TD)
- Over the top femoral position ACL reconstruction (OTT) length = 8.25 + (0.85 × TD) + tibial tunnel
- Marcacci technique (MT) length = 29.47 + (1.43 × TD) + tibial tunnel

Since the anatomic anterolateral ligament reconstruction 3D measurement showed a poor interobserver reliability, the formula obtained with linear regression was considered inaccurate and not included here. All formulas must be intended in mm.

### Accuracy of the formulas

To test the accuracy of the formulas, 3D measurements performed on the MRI and calculated with the formulas were compared with a paired *t*-test for every 3D parameter included in the study, showing no difference between the groups: intrarticular length of ACL graft *p* = 0.98, outside-in femoral tunnel length *p* = 0.97, outside-in anatomic ACL reconstruction *p* = 0.94, Saragaglia/Yamaguchi techniques *p* = 0.97, lateral extrarticular tenodesis (anatomical femur) *p* = 0.98, lateral extrarticular tenodesis (non-anatomical femur) *p* = 0.99, over the top femoral position ACL reconstruction *p* = 0.97, Marcacci technique *p* = 0.9721.

**Fig. 4:**
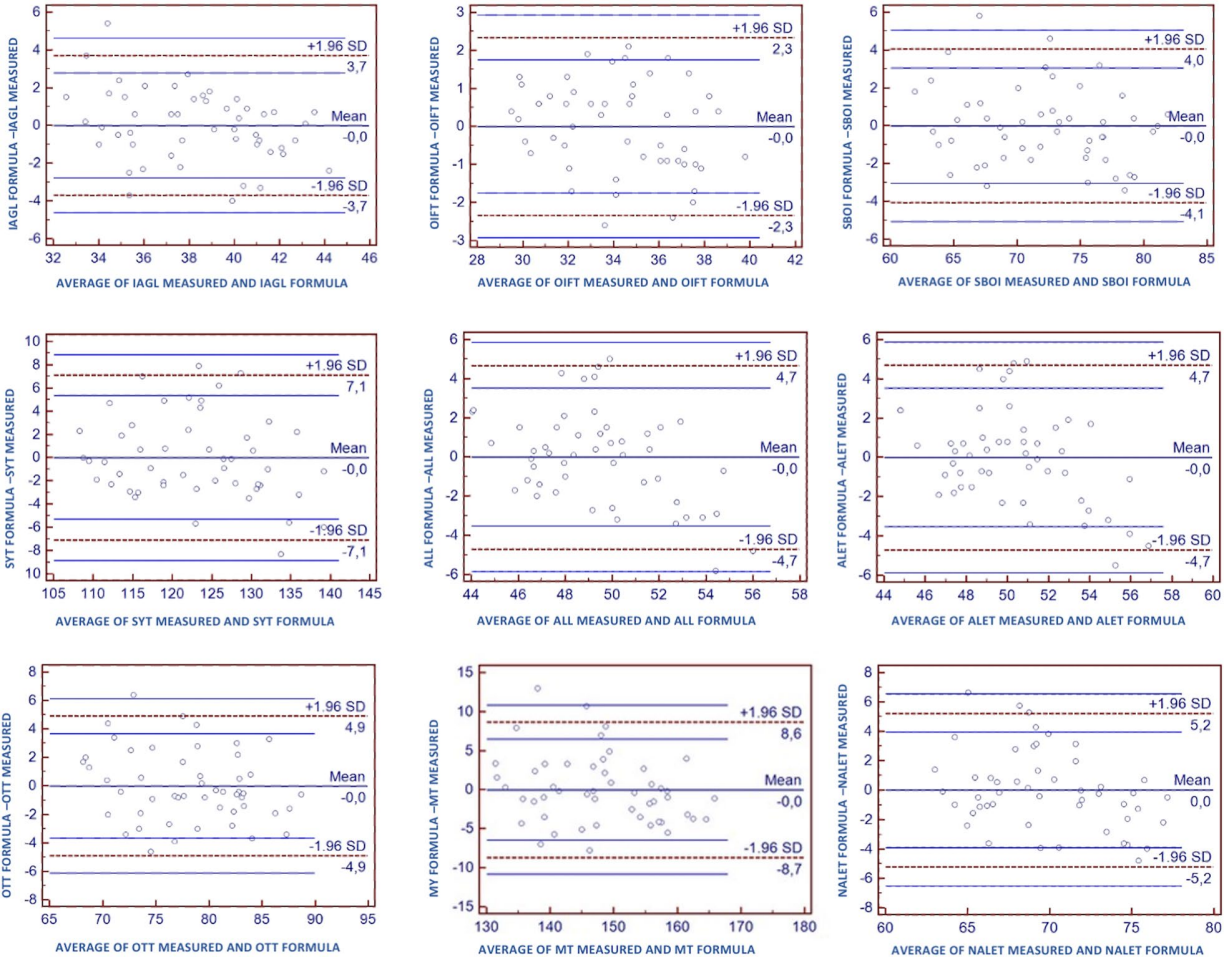
Bland–Altman plots to compare 3D measurements performed on the MRI and calculated with the formulas. *IAGL* Intrarticular length of the graft, *OIFT* outside-in femoral tunnel, *SBOI* single bundle outside-in anatomic ACL reconstruction, *ALL* anatomic anterolateral ligament, *ALET* modified Lemaire lateral extrarticular tenodesis, anatomical on the femur, *SYT* Saragaglia and Yamaguchi technique, *OTT* over the top femoral position ACL reconstruction, *MT* Marcacci technique, *NALET* modified Lemaire lateral extrarticular tenodesis, *SD* standard deviation

The 3D measurements performed on the MRI and calculated with the formulas were also compared with the Bland–Altman plot (Fig. 4). The Bland–Altman plot [23, 24], or difference plot, is a graphical method to compare two measuring techniques. With this method, the differences between the two techniques are plotted against the averages of the two techniques. In this study, since the formulas were obtained from the 3D measurements performed on the MRI, the means of the two measuring techniques are identical and this is demonstrated by the mean difference being 0.0 on the plots (central blue line on Fig. 4).

Another advantage of the Bland–Altman plot in this type of study is that investigators can interpret the limits of agreement to assess whether the agreement is acceptable, based on what is clinically important or not. The limits of agreement are expected to include about 95% of the differences observed in the future. In this study, the acceptable range of agreement was defined a priori at ± 5 mm for shorter measurements (IAGL, OIFT, SBOI, ALL, OTT), from −3 mm to +20 mm for longer measurements (SYT and MT), and from 0 mm to +15 mm for lateral extrarticular tenodesis (ALET and NALET). In fact, from the clinical point of view, an over- or under-estimation of 5 mm can be acceptable for shorter measurements (i.e., for a single bundle out in femoral tunnel ACLR revision to minimize the graft-tunnel mismatch). On the other hand, for longer reconstruction techniques (i.e., Saragaglia, Marcacci, or Yamaguchi) or lateral extrarticular tenodesis, a minimal underestimation of the graft length can be accepted from the formula, but an overestimation of the length does not represent a clinical problem in the surgical setting: i.e., a surgeon does not want a graft shorter than needed, but can always cut the remnant of a longer graft. Based on the Bland–Altman plots, the formulas for the shorter ACLR revision measurements (IAGL, OIFT, SBOI, ALL, OTT) were considered accurate enough. On the other hand, according to the needs of the surgeons, 7 mm were added to the formulas for longer ACLR revision measurements (SYT and MT) and 6 mm to the formulas for lateral extrarticular tenodesis (ALET and NALET). In addition, the decimals of all formulas were rounded for easier clinical application. The final formulas were:

- Intrarticular length of ACL graft (IAGL) = (0.46 × TD)
- Outside-in femoral tunnel length (OIFT) = 1 + (0.42 × TD)
- Outside-in anatomic ACL reconstruction (SBOI) length = 2 + (0.84 × TD) + tibial tunnel
- Length of Saragaglia/Yamaguchi techniques (SYT) = 22 + (1.3 × TD) + tibial tunnel
- Lateral extrarticular tenodesis anatomical femur (ALET) length = 21 + (0.42 × TD)
- Lateral extrarticular tenodesis non-anatomical femur (NALET) length = 27 + (0.59 × TD)
- Over the top femoral position ACL reconstruction (OTT) length = 8 + (0.85 × TD) + tibial tunnel
- Marcacci technique (MT) length = 36 + (1.43 × TD) + tibial tunnel

Please note that TD, tibial tunnel, and the result of the formula are all expressed in millimeters (mm).

## Discussion

The main findings of the present study were as follows (1) the intra and interobserver reliability of linear 2D measurement on standard 2D MRI sequences was high, with the transepicondylar distance (TD) showing the highest reproducibility and repeatability; (2) the intra and interobserver reliability of complex/curved 3D measurements on 3D MRI was lower than 2D measurements, but acceptable for all measurements, except for anatomic anterolateral ligament reconstruction; (3) a strong linear correlation was found between most 2D MRI measurements and 3D measurements, except for patellar tendon length; (4) the TD showed the strongest correlation with 3D measurements; and (5) the authors were able to extract accurate formulas to calculate the length of the graft required for different ACLR revision techniques from the TD measured on standard 2D MRI.

The position of the present study in the existing sports medicine literature is rather unique. Some papers have studied the correlation between morphometric parameters in arthritic knees (measured preoperatively on different imaging techniques or intraoperatively) and the final total knee replacement implant, in terms of size or rotation [25–31].

Some other papers have investigated whether anthropometric parameters or MRI measurements were able to predict the length and thickness of hamstring graft for ACLR [31–35]. However, few of these studies were able to describe reliable formulas for the prediction of hamstring length and diameter [36].

As previously mentioned, ACLR revision surgery cannot be standardized as primary ACLR, and for this reason there is an increasing interest among sports medicine surgeons regarding the preoperative planning, with the goal of avoiding intraoperative complications. Grasso et al. compared 3D MRI and 3D computed tomography (CT) scans in 24 patients diagnosed with a failed ACLR, to establish an accurate MRI protocol as an alternative to 3D-CT scans. The authors found that a high-resolution 3D turbo spin echo proton density MRI sequence could quantitatively evaluate the location and orientation of previous bone tunnels for routine postoperative assessment, presurgical planning, and outcome evaluation [37]. With the same goal of improving preoperative planning in ACLR revision surgery, Kitamura et al. found that 3D printed models of the knee were a useful addition to CT scans for sports medicine orthopedic fellows during the planning of ACLR revision surgery [38]. Most of these studies dealing with preoperative planning of ACLR revision have focused on accurately determining the size, location, and orientation of previous bone tunnels; this is certainly one of the first aspect to consider when approaching ACLR revision surgery, together with ruling out possible comorbidities responsible for ACL failure (i.e., lower limb malalignment, meniscal deficiency, previously overlooked concomitant knee instabilities). All these aspects are crucial to decide between a single-stage or a two-stage ACLR revision, and to choose the appropriate surgical technique. On the tibial side, enlarged (> 16 mm) or malpositioned previous tibial tunnels putting at risk tibial fixation, stability, and healing of the graft are a strong indication for a staged ACLR revision. Conversely, on the femoral side, if a new tunnel performed with standard technique (i.e., antero-medial or outside-in drilling) put at risk fixation or stability of the graft, “non-conventional” techniques, such as the “over-the-top” can be performed to avoid the existing femoral tunnel [11, 39].

In addition, based on the recommendations of the Anterolateral Complex consensus group [4], ALL reconstruction or lateral extrarticular tenodesis (LET) should be associated to the ACLR in the revision setting. For all these reasons, also considering other unconventional techniques seems reasonable in ACLR revisions [40]. These techniques (i.e., Marcacci, Yamaguchi, Saragaglia) have been described as “unconventional” throughout the whole manuscript because they are not standard procedures in most surgeons’ hands for primary ACLR, but they can be performed in both primary and revision settings.

In the Marcacci technique, the hamstring tendons are harvested preserving the distal insertion. The graft is then passed through a tibial tunnel and “over the top” on the femoral side using a lateral incision to the distal femur. The graft is fixed just proximal to the lateral femoral condyle with two Richard’s staples, with the knee flexed at 90° and the foot externally rotated. The remnant of the graft is then passed under the iliotibial band and fixed with one staple at the level of the Gerdy’s tubercle [41]. In 2009, Marcacci et al. evaluated the outcomes of 54 consecutive high-level athletes treated with their technique at a 10–13 year follow-up. The IKDC score demonstrated good or excellent results in 90.7% of the patients, and the radiographic evaluation demonstrated progressive joint narrowing only for the 20 patients who received concomitant medial meniscus surgery [12].

In 2006, Yamaguchi et al. described an iliotibial band (ITB) ACLR with combined anterolateral plasty. A 25 cm longitudinal incision is performed on the lateral femur. A 22 cm-long strip of the ITB is harvested, leaving the tibial insertion attached on the Gerdy’s tubercle. A 7.5 mm femoral tunnel is then drilled in an outside-in fashion. A 7.5 mm tibial tunnel is then drilled. The graft is passed deep to the LCL and through the femoral and tibial tunnels. With the knee at 90° of flexion and the foot externally rotated, the graft is sutured to the LCL and the periosteum of the lateral femoral condyle. Then, with the knee at 30° of flexion and the foot externally rotated, the graft is sutured to the periosteum around the outlet of the tibial tunnel. The authors described good long-term outcomes, with a mean KT-1000 side-to-side difference of 3.5 mm at 24 years follow-up [13].

A technique very similar to Yamaguchi’s was described in 2013 by Saragaglia et al. The hamstrings are harvested and left attached distally on the proximal anteromedial tibia. At the level of the intrarticular portion, the semitendinosus is doubled. Tibial and femoral full tunnels are drilled with an outside-in technique. The graft is then passed into the knee through the tunnels, exiting the lateral femur. A second lateral skin incision is made on the proximal tibia for the anterolateral plasty. The tail of the graft is tensioned towards Gerdy’s tubercle and attached to the fascia lata with five or six large absorbable sutures and the foot in a neutral position. Again, the authors described good outcomes with this technique, with no pivot shift in 75% of the cases [14].

The graft required for all these reconstruction techniques is much longer than standard ACLR procedures. Both Marcacci and Yamaguchi underlined in their papers the importance of having a long enough graft (20 cm according to Marcacci and 22 cm for Yamaguchi) [13, 41]. However, not all knees are the same size and an absolute value (20 or 22 cm) for graft length cannot be applied to every patient. Indeed, in our study population, a graft length difference of up to 4 cm was found between taller and shorter patients. One of the goals of this study was to provide the surgeons with an easy formula to determine preoperatively the required length of the graft, helping surgeons to harvest hamstrings and ITB or request an adequately long allograft to the tissue bank. In addition, some of the formulas extracted were modified according to the surgeons’ needs and to avoid the complication of a too-short graft in all patients. This was done for those procedures where having a short graft could compromise the surgery: Marcacci, Yamaguchi, Saragaglia, and lateral extrarticular tenodesis. The formulas were not modified when the clinical needs required the most precise estimation of the graft length. For instance, this scenario applies when trying to avoid a graft-tunnel mismatch: i.e., during outside-in femoral tunnel or femoral over the top BPTB single bundle ACLR revisions.

Regarding lateral extrarticular tenodesis (LET), two different techniques were included: (1) an anatomical LET (ALET), with the femoral fixation located at the anatomical insertion of the ALL [19] and (2) a non-anatomical LET (NALET) with a more proximal and posterior fixation on the femur [21]. Between the many LETs described in the literature, since no technique has shown to be superior over the others, the longest and shortest LET procedures were included in the present study.

This study is not without limitations. First, the data were obtained from MRI measurements and not cadaveric knees. However, the number of specimens required would have been too large for the study to be performed on cadavers, and the reliability of MRI measurements on DICOM viewers is well established. In addition, the design of the study entailed calculating the graft length with a formula from a simple 2D MRI measurement, and this would not have been possible in cadaveric specimens. However, a spin-off study comparing the data extracted from the formulas with real reconstructions (during surgeries or on cadaveric specimens) would be advisable. A second limitation is related to the ethnicity of the population studied: all patients included were Caucasian and significant morphological differences have been described among patients of different races [18]. As a last limitation, the tibial tunnel is a missing datum from some of the formulas. However, the tibial tunnel length can be varied by the surgeon, based on where the tibial tunnel distal aperture is placed. For this reason, the authors decided to exclude the tibial tunnel length from the measurements. Also, being able to slightly adjust the tibial tunnel length is an advantage for the surgeon, who has an additional degree of freedom during surgery to avoid graft-tunnel mismatch.

## Conclusion

In conclusion, the transepicondylar distance (TD) showed the highest repeatability and reproducibility, as well as the strongest correlation with 3D measurements. Formulas were created from the TD and based on the clinical needs, for the following procedures: ACLR revision techniques (Marcacci, Saragaglia, Yamaguchi, “over the top” femoral fixation, outside-in femoral drilling) and two lateral extrarticular tenodesis (LET) techniques (anatomical and non-anatomical). The formulas extracted proved to be accurate and can be used by surgeons in ACLR revision pre-operative planning, graft harvesting and allograft selection.

## Data Availability

All data are available at the corresponding author upon request.

## Declarations

### Patient involvement

Patients or the public were not involved in the design, or conduct, or reporting, or dissemination plans of our research.
